# Cerebellar Expression of the Neurotrophin Receptor p75 in Naked-Ataxia Mutant Mouse

**DOI:** 10.3390/ijms17010115

**Published:** 2016-01-15

**Authors:** Maryam Rahimi Balaei, Xiaodan Jiao, Niloufar Ashtari, Pegah Afsharinezhad, Saeid Ghavami, Hassan Marzban

**Affiliations:** 1 Department of Human Anatomy & Cell Science, Faculty of Health Sciences, College of Medicine, University of Manitoba, Winnipeg, MB R3E 0J9, Canada; rahimibm@myumanitoba.ca (M.R.B.); jiaox3@myumanitoba.ca (X.J.); ashtarin@myumanitoba.ca (N.A.); umafshar@myumanitoba.ca (P.A.); saeid.ghavami@umanitoba.ca (S.G.); 2 Health Policy Research Center, Shiraz University of Medical Science, Shiraz 713484579, Iran

**Keywords:** Purkinje cell, p75NTR, gene expression, *nax* mutant

## Abstract

Spontaneous mutation in the lysosomal acid phosphatase 2 (Acp2) mouse (*nax*—naked-ataxia mutant mouse) correlates with severe cerebellar defects including ataxia, reduced size and abnormal lobulation as well as Purkinje cell (Pc) degeneration. Loss of Pcs in the *nax* cerebellum is compartmentalized and harmonized to the classic pattern of gene expression of the cerebellum in the wild type mouse. Usually, degeneration starts in the anterior and posterior zones and continues to the central and nodular zones of cerebellum. Studies have suggested that the p75 neurotrophin receptor (NTR) plays a role in Pc degeneration; thus, in this study, we investigated the p75NTR pattern and protein expression in the cerebellum of the *nax* mutant mouse. Despite massive Pc degeneration that was observed in the *nax* mouse cerebellum, p75NTR pattern expression was similar to the HSP25 pattern in *nax* mice and comparable with wild type sibling cerebellum. In addition, immunoblot analysis of p75NTR protein expression did not show any significant difference between *nax* and wild type sibling (*p* > 0.5). In comparison with wild type counterparts, p75NTR pattern expression is aligned with the fundamental cytoarchitecture organization of the cerebellum and is unchanged in the *nax* mouse cerebellum despite the severe neurodevelopmental disorder accompanied with Pc degeneration.

## 1. Introduction

Lysosomal acid phosphatase 2 (Acp2) is a key enzyme in cerebellar development [1,2,3], and it is implicated in a variety of neurological disorders including progressive supranuclear palsy [4] and juvenile neuronal ceroid lipofuscinosis, also known as Batten disease (a disease induced by mutations in the lysosomal protein ceroid-lipofuscinosis neuronal 3 (CLN3)) [5]. Acp2 hydrolyzes orthophosphoric monoesters into phosphate and alcohol. Mounting evidence indicates that a spontaneous autosomal recessive mutation in Acp2 is responsible for occurrence of a neurocutaneous disorder that is correlated with abnormal skin and cerebellar development, and is also called naked-ataxia (*nax*) mice [2,3]. In addition to the *nax* mouse, several studies have used the Acp2 transgenic animal model to investigate the role of Acp2 deficiency in development of the nervous system, particularly in the cerebellum [2,3,6,7].

According to differential gene expressions, the cerebellum has been divided into four transverse zones including: (1) the anterior zone (AZ: lobules I–V); (2) the central zone (CZ: lobules VI–VII: with two sub-zones—see [8,9]); (3) the posterior zone (PZ: lobules VIII + dorsal lobule IX); and (4) the nodular zone (NZ: ventral lobule IX + lobule X) (e.g., [1,10,11]). In addition, each zone is subdivided mediolaterally into parasagittal stripes that are symmetrical about the midline [10,12]. Much of the cerebellar patterning seems to have been built on the Purkinje cell (Pc) scaffold [13] because cerebellar cell types, functions and their afferent and efferent roles are aligned in a zone and stripe pattern [14,15,16].

Our laboratory has recently revealed that Acp2 plays a pivotal role in development of the cerebellum through expression in the caudal mesencephalon and diffusely in the developing cerebellum. It is further expressed in the adult mouse cerebellum by a subset of Pcs that are aligned in the zebrin II zone and stripe pattern [1]. Another important finding in the *nax* mouse was the pattern of the gene expression in the cerebellum which altered particularly in the AZ and CZ [2]. The density and population of Pcs decreased significantly in response to an unknown cell death mechanism with aging of the *nax* cerebellum. We have previously shown that probably Pc death in *nax* cerebellum is not associated with the classic apoptosis signaling pathway [2], as observed in the Pc degeneration (Pcd) mouse [17,18].

On the other hand, neurotrophins are highly involved in processes related to neuronal survival, development and function [19,20]. Neurotrophins have been reported to regulate neuronal apoptosis, a process that is critical in the developing brain and also in a variety of neurodegenerative diseases [21]. During development, nerve growth factor (NGF) mediates a variety of neuronal functions through activation of the p75NTR [22]. Depending on the physiological, pathophysiological or developmental timing of the brain, it has been proposed that p75NTR plays a dual role in cell survival or death [23,24].

In pathological conditions, upregulation of p75NTR and replacement of this receptor is the key in controlling the numerous processes that are necessary for nervous system recovery [25]. Currently, p75NTR is an important potential target for pharmacological control of neurotrophin activity [25]. In this respect, we aimed to examine whether p75NTR is altered with progressive Pc loss in a *nax* model of neurodevelopmental and neurodegenerative diseases.

## 2. Materials and Methods

### 2.1. Animal Maintenance and Tissue Processing

In the current study, animal procedures were based on institutional regulations and the *Guide to the Care and Use of Experimental Animals* from the Canadian Council for Animal Care (CCAC) and University of Manitoba Animal Care Committee (ACC). All efforts were made to minimize the number of animals used and the animals were treated in a humane manner.

*Nax* mutant embryo samples were obtained from the Institute of Human Genetics in the University Medical Center, Georg-August University, Goettingen, Germany. A colony was established in the Genetic Model Center at the Faculty of Health Sciences, University of Manitoba by breeding mice (C57BL/6) heterozygous for the *nax* mutation. Animals were maintained at room temperature (RT) and relative humidity (18–20 °C, 50%–60%) on a 12-h light-dark cycle with free access to food and water.

Phenotypically, *nax* mutant mice were easily recognized from their littermate counterparts based on the delayed appearance or lack of fur over the body, smaller size and ataxia. In order to confirm the genotypes, PCR was carried out according to Mannan *et al.* [3], using the following primers: Acp4F (GCACTCTGTGCCTTCTCCAT) and Acp4R (CTGGGAGATTTGGGCAACTA). Samples were prepared based on our previously published study [2]. Briefly, adult and postnatal developing mice from P4 to P28 (wild type (wt); *n* = 18 and *nax*; *n* = 18) were transcardially perfused and brains post-fixed in 4% paraformaldehyde. Then, cerebella were cryoprotected in sucrose series and optimal cutting temperature to take sections at 30 µm in transverse and sagittal planes.

### 2.2. Antibodies

In this study, we used two different anti-calbindin antibodies: rabbit polyclonal anti-calbindin D-28K antiserum (anti-CaBP, diluted 1:1000, Swant Inc., Bellinzona, Switzerland) and mouse monoclonal anti-calbindin (anti-CaBP, diluted 1:1000, Swant Inc. [26,27]). These antibodies have been reported as appropriate tools for Pc-specific staining (e.g., [28]).

For the p75 neurotrophin receptor (p75NTR), rabbit polyclonal antibody, was purchased from Cell Signaling Technology, Danvers, MA, USA (diluted 1:1000). P75NTR is expressed in some cerebellar Pcs [29].

For heat shock protein (HSP) 25, expressed in a subset of Pcs (for details see [2,30]), anti-small HSP25, a rabbit polyclonal antibody, was obtained from StressGen, Victoria, BC, Canada (diluted 1:1000).

For the paired box protein (Pax), anti Pax6 was used for mouse monoclonal antibody (Developmental Studies Hybridoma Bank, Department of Biology, University of Iowa, Iowa City, IA, USA) and it was diluted 1:1000. This transcription factor is expressed in developing cerebellar granule cells [31].

### 2.3. Immunohistochemistry

For section immunohistochemistry, we used peroxidase and double-labelled fluorescent immunohistochemistry as previously reported [1,8]. In brief, peroxidase immunohistochemistry was performed by washing the cerebellar sections with PBS and then, endogenous peroxidase activity was halted using 0.3% H_2_O_2_. Samples were incubated in blocking solution and primary antibody overnight at room temperature (RT). After washing with PBS, tissue samples were incubated in secondary antibodies at RT and then visualized by diaminobenzidine (DAB) as the chromogen. For negative control samples, similar as mentioned above, addition of primary antibody was replaced by a suitable IgG isotype. Sections were washed with PBS and peroxidase activity was measured by using DAB. For anti-HSP25 immunohistochemistry, biotinylated secondary antibody was applied.

For double immunofluorescence, samples were co-incubated in primary antibodies overnight (RT) and then incubated in Alexa Fluor 488 goat anti-mouse IgG and Alexa Fluor 594 goat anti-rabbit IgG for 1 hat RT. Samples were then washed in PBS and coverslipped with Fluorsave.

### 2.4. Western Blotting

In order to measure the expression of p75NTR at the protein level, Western blot analysis was conducted using a previously described protocol (e.g., [1]). Briefly, cerebellar tissue was homogenized at different time intervals in postnatal (P) stage (P5, 9 and 18) from wt and *nax* mice of three different litters at each age (*n* = 9 for both *nax* and wt mice). Then, protein concentrations were assessed using a commercial BCA kit. Diluted samples were electrophoresed in loading buffer and transferred to an Immobilon-P transfer membrane (Millipore, Mississauga, ON, USA). For immunostaining, membranes were blocked for 1 h in 5% skim milk in TBS + 0.1% Triton X-100 (PBST). Membranes were incubated with primary antibody (rabbit polyclonal anti-p75NTR was diluted in PBST) at 4 °C for 18 h with gentle agitation. Membranes were incubated for 1 h with HRP conjugated goat anti-rabbit IgG and diluted 1:6000 in TBST. Binding was assessed using the Enhanced Chemiluminescence (ECL) protocol on Scientific Imaging Film.

### 2.5. Preparation of the Figures

In the case of bright field microscopy, a Zeiss M-2 microscope was applied and images were taken using Zen software. For fluorescence microscopy, a Zeiss Lumar.V12 stereomicroscope was applied to record the images of cerebellar sections using AxioVision 4 software. For high magnification, fluorescence microscopy a Zeiss Z2 Imager with Zen software was used to record the images. After cropping the images, they were corrected for brightness and contrast, and finally assembled into montages using Adobe Photoshop CS5 Version 12.

### 2.6. Statistical Methods

Western blot data are shown as the mean ± standard error of mean (SEM) and they were analyzed using the Kruskal-Wallis test followed by Dunnett’s multiple comparison test using Mann-Whitney test. We considered *p* > 0.05 as non-significant result. Statistical analysis was done using SPSS software (Version 18 SPSS Inc. Chicago, IL, USA).

## 3. Results

### 3.1. Expression Pattern of p75NTR in Adult Mouse Cerebellum

**Figure 1:**
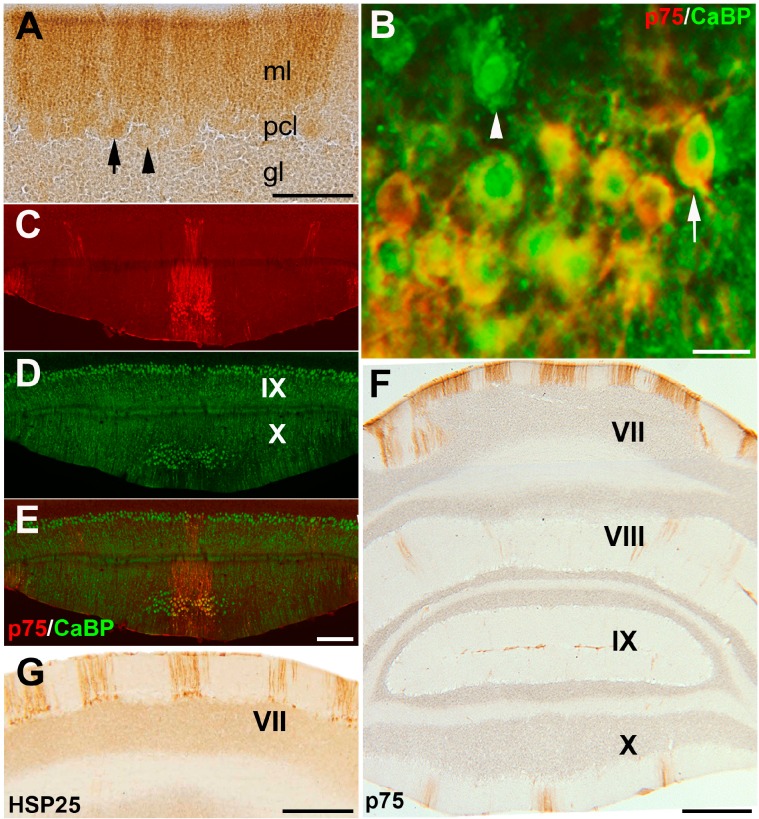
Frontal sections of an adult mouse cerebellum immunostained with p75NTR, CaBP and HSP25 at P28. The lobules are shown by Roman numerals. (**A**,**B**) The cerebellar cortex immunoperoxidase stained by p75NTR shows that the Pc somata in the Purkinje cell layer and dendrites in the molecular layer are immunoreactive (arrow; black in A, and white in B). Some Pc somata expressing p75NTR are weak (A, arrowhead) or absent (B, arrowhead); (**C**–**E**) Double immunostaining of a frontal section at lobule IX and X using p75NTR (red) and CaBP (green). Stripes of immunoreactive Pcs are clear in the vermis; (**F**) Immunostaining of a frontal section at the cerebellum using p75NTR. Immunoreactivity is not present in the AZ and PZ. Stripes of immunoreactive Pcs are clear in the vermis of the CZ (VII) and NZ (X); (**G**) Immunostaining of a frontal section through the lobule VII using HSP25. Stripes of immunoreactive Pcs are clear in the vermis of the CZ. Abbreviations: ml, molecular layer; Pcl, Purkinje cell layer; gl, granular layer.Scale bars: **A** = 100 µm; **B** = 20 µm; **E** = 250 μm (applies to **C**–**E**); **F** = 500 µm; **G** = 250 µm.

A transverse section at the cortex of adult animal cerebellum showed that p75NTR is strongly (arrow) or weakly (arrowhead) expressed in Pc somata in the Pc layer as well as dendrites in the molecular layer (Figure 1A). In order to confirm p75NTR expression in Pcs, a double immunostaining with CaBP (a specific marker for Pcs) was performed and showed p75NTR localization in Pcs (Figure 1B). Expression of the p75NTR is restricted to a subset of Pcs in the cerebellar cortex and alternates with p75NTR immuno-negative Pcs (Figure 1C–E). Low magnification of transverse sections at the mouse cerebellum revealed that p75NTR express in parasagittal stripes symmetrical about the midline (Figure 1F). The immunoreactivity pattern of p75NTR is strongly present in the CZ (lobule VI/VII) and NZ (IXb/X), but not in the AZ (I–V) and PZ (VIII/IXa) of the adult cerebellum (Figure 1F). P75NTR expression is similar to the pattern of HSP25 expression in mouse cerebellum, localized to the CZ (Figure 1G) and NZ (e.g., [2]).

### 3.2. Pattern Expression of p75NTR in wt and nax Cerebellum at P4 and P6

**Figure 2:**
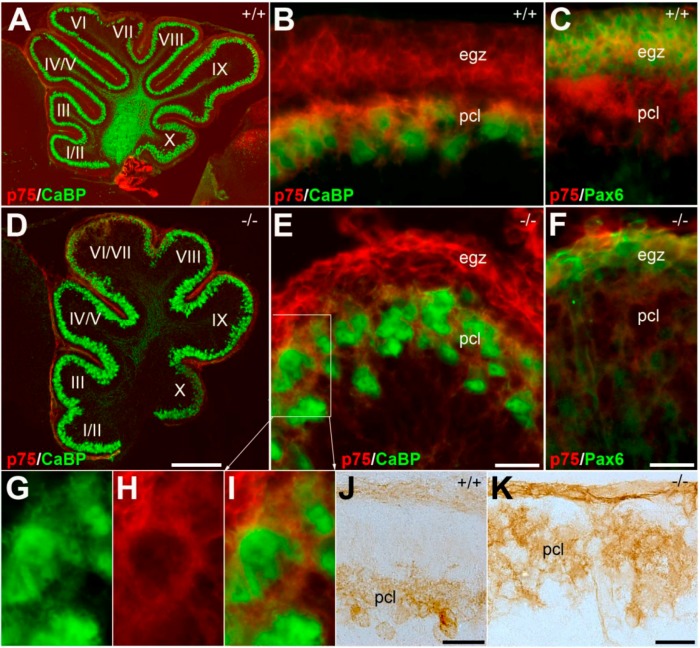
Sagittal sections of P4 wt^+/+^ (**A**–**C**) and *nax*^−/−^ (**D**–**I**) and sagittal sections of P6 wt (**J**) and *nax* (**K**) cerebella immunostained with p75NTR, CaBP and Pax6. The lobules are shown by Roman numerals. (**A**–**C**) The wt cerebellum shows normal lobules (**A**) with expression of p75NTR (red) in the entire egz (external germinal zone)and dispersing through the Pc layers. A higher magnification of “A” shown in “B” and Pax6 immunostaining is indicated egz in “C”; (**D**–**F**) The *nax* cerebellum shows a small cerebellum with underdeveloped lobulation (**B**). P75NTR expression (red) is present in the underdeveloped egz and in the developing Pcl (**B**). Immunostaining with Pax6 shows the thin layer of the egz in the *nax* cerebellum (**F**); (**G**–**I**) Magnified views of white box in E showing CaBP (**G**), p75NTR (**H**), and merged image (**I**); (**J**–**K**) The sagittal sections immunoperoxidase stained by p75NTR shows immunoreactivity of the Pc in wt (**J**) and *nax* (**K**) cerebella. Abbreviations: egz, external germinal zone; Pcl, Purkinje cell layer. Scale bars: **D** = 500 µm (applies to **A** and **D**); **E** = 50 µm (applies to **B** and **E**); **F** = 40 μm (applies to **C** and **F**); **J** = 50 µm; **K** = 50 µm.

P75NTR expression was studied in midsagittal and frontal sections of *nax* and wt sibling cerebellum to determine distribution of p75NTR in developing cerebellar cortex at P4 and P6. Double immunohistochemistry with CaBP revealed that p75NTR was widely expressed in midsagittal sections throughout the cerebellar cortex of wt and *nax* mice at P4 (Figure 2A,D). At a higher magnification, it is shown that p75NTR expression is localized in the developing Pc layer in the wt sibling (Figure 2B) and *nax* (Figure 2E) cerebellar cortex , with strong expression in the Pc membrane and weakly in the cytoplasm (Figure 2G–I). In addition, p75NTR is expressed in the area superficial to the developing Pc layer, at the location of the external germinal zone (egz) (Figure 2B,E). Double staining with Pax6, a marker for granule cell precursors confirmed that p75NTR is expressed in the EGZ (Figure 2C,F). A sagittal section at the cerebellar cortex immunoperoxidase stained by p75NTR shows immunoreactivity of the Pc in wt (J) and *nax* (K), in which Pcs are multilayered in molecular layers.

**Figure 3:**
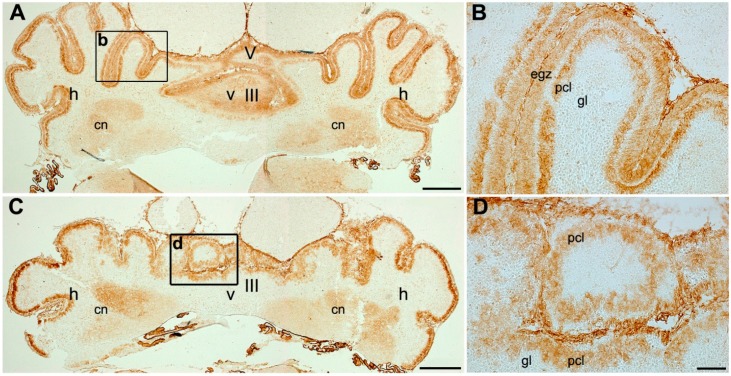
Frontal sections at the cerebellum of wt (**A**,**B**) and *nax* (**C**,**D**) at P6 immunoperoxidase stained with p75NTR. (**A**,**B**) The wt cerebellum shows normal vermis (v) and hemisphere (h) lobulation (**A**) with uniform expression of p75NTR in the entire cerebellar cortex. A higher magnification of black box (b) in “**A**” indicates localization of p75NTR expression in Pcl and EGZ; (**C**,**D**) Underdeveloped *nax* cerebellum exhibits uniform p75NTR expression in putative vermis and in the hemisphere. Magnified views of black box (d) in “**C**” showing p75NTR in multilayer Pc and thin or lack of EGZ. The p75NTR expression shows the outline of cerebellar nuclei (cn) both in wt (**A**) and *nax* (**C**) sections. Abbreviations: egz, external germinal zone; Pcl, Purkinje cell layer; h, hemisphere; cn, cerebellar nuclei; gl, granular layer. Scale bars: **A** and **C** = 500 µm; **D** = 100 µm (applies to **B** and **D**).

In order to understand whether p75NTR expression is in uniform or stripe pattern to further study the possible alteration in *nax* in comparison with wt sibling, the early postnatal cerebellar cortex was immunoperoxidase stained by p75NTR at P6 (Figure 3). P75NTR is expressed in the cerebellar cortex and weakly in cerebellar nuclei, probably due to Pc terminal axon in cerebellar nuclei region (Figure 3A). The pattern expression of p75NTR is uniform in the entire cerebellar cortex (Figure 3A) and clearly in Pc layer and external germinal zone, but not in the granule cell layer (Figure 3B). Despite the underdeveloped *nax* cerebellar vermis and the atypical lobulation, the pattern is comparable to wt sibling (Figure 3C). The p75NTR is expressed uniformly in the cortex of the *nax* cerebellum where the multilayer Pcs are invaded to the molecular layer accompanied with thin or no external germinal zone and granule cells (Figure 3D).

Overall, the pattern of p75NTR expression was normal at P4/P6 in *nax* mice, even with the small size of the cerebellum, the underdeveloped lobulation and corticogenesis with abnormal Pc multilayer, and a thin layer or lack of EGZ.

### 3.3. Pattern Expression of p75NTR in wt and nax at P22

**Figure 4:**
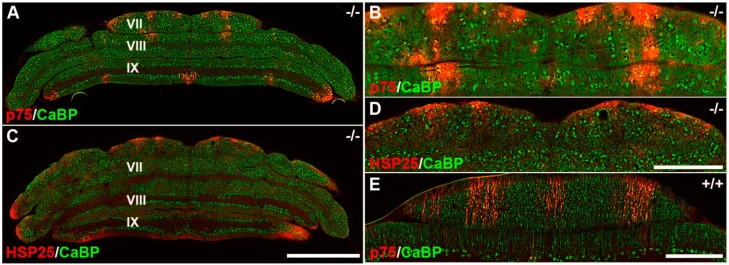
Transverse sections of *nax*^−/−^ (**A**–**D**) and wt sibling cerebella at P22 immunostained with p75NTR, CaBP and HSP25. (**A**,**B**) P75NTR immunostaining shows an array of parasagittal stripes in the CZ and NZ of the *nax* cerebellum, with high magnification of the CZ shown in **B**; (**C**,**D**) HSP25 immunostaining of the *nax* cerebellum shows parasagittal stripes in the CZ and NZ, **D** is a higher magnification of **C**; (**E**) At a high magnification, p75NTR expression is shown in the CZ of the wt^+/+^ sibling. Scale bars: **C** = 1 mm (applies to **A** and **C**); **D** = 500 µm (applies to **B** and **D**); **E** = 250 μm.

Cellular death is prominent in the *nax* cerebellum [2] and, p75NTR has been suggested to be involved in Pc degeneration [24]. To determine whether p75NTR pattern expression is altered in *nax* cerebellum with severe cerebellar cell death, we examined the p75NTR pattern expression in *nax* and wt siblings and compared it with HSP25 at P22 (Figure 4). Double immunostaining with p75NTR and CaBP through the CZ and NZ of the *nax* mutant cerebellum revealed an array of parasagittal stripes (Figure 4A), which was shown at a higher magnification of lobule VI (Figure 4B). P75NTR expression in CZ and NZ was also compared with HSP25 expression in the CZ and NZ in the *nax* cerebellum. Because both p75NTR and HSP25 are rabbit polyclonal antibodies, double staining was not performed. Consequently, we used serial section of the *nax* cerebellum to compare the p75NTR and HSP25 expression pattern. P75NTR was similar to that of HSP25 in the CZ and NZ (Figure 4C) and a higher magnification of CZ is shown in Figure 4D. Double immunofluorescence staining with p75NTR and CaBP has shown the presence of p75NTR stripes in lobule VII of wt siblings (Figure 4E).

### 3.4. Protein Expression of p75NTR in wt and nax Cerebellum

**Figure 5:**
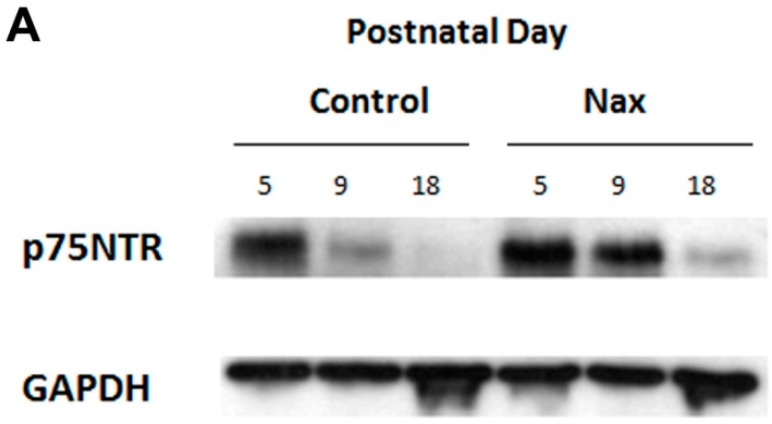

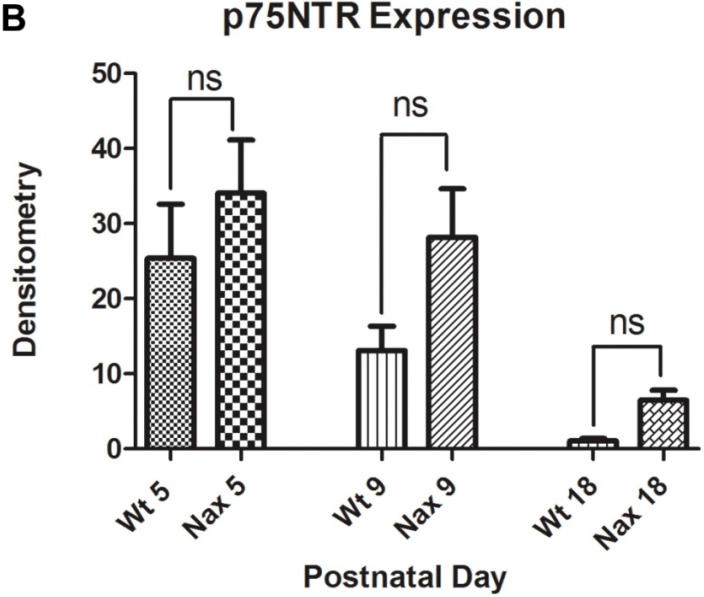
Western blot analysis of p75NTR expression during cerebellar development at P5, P9 and P18. This experiment was repeated over three different litters for each postnatal days in wt and *nax* siblings (P5, P9 and P18, wt; *n* = 9 and *nax*; *n* = 9). (**A**) Immunoblots of total cell lysate from wt sibling and *nax* mouse cerebellum indicate significant down-regulation in the wt sibling and also in the *nax* cerebellum from early postnatal development to P18. However, there is no considerable difference in p75NTR expression between the wt and *nax* cerebellum, despite the higher p75NTR expression in *nax* compared with wt. (*p* > 0.05). Protein loading was confirmed using GAPDH (*n* = 18); (**B**) The data in the bar graph are presented as the mean ± SEM, and statistical analysis was performed using Kruskal-Wallis test followed by Dunnett’s multiple comparison test using Mann-Whitney test. Abbreviation: ns, not significant.

To determine whether the protein expression of the p75NTR is altered in *nax* and wt siblings, we used western blot at P5, 9 and 18. Western blot analysis indicates that p75NTR protein expression gradually decreases as development proceeds in both *nax* and wt siblings. However, despite the apparent higher expression of p75NTR in the *nax* cerebellum, or probably delayed down regulation of p75NTR protein expression, there is no significant difference between *nax* and wt sibling cerebellum at any of these ages (Figure 5A,B) (*p* > 0.05).

## 4. Discussion

In the current work, we examined p75NTR expression in postnatal *nax* mutant mice cerebellum to determine its pattern and protein expression and to identify whether abundant Pc degeneration in *nax* cerebellum is associated with changes in the p75NTR expression. The p75NTR expression is localized in the EGZ and Pc layer in early postnatal development, while it is patterned in parasagittal stripes in the CZ and NZ of the adult mouse cerebellum, similar to HSP25 pattern expression. Although the *nax* cerebellum has severe abnormalities and Pc degeneration [2] there seems to be no alteration in the p75NTR pattern and protein expression in the *nax* cerebellum compared with the wt sibling.

The cerebellar compartmentation has a fundamental cytoarchitecture organization in which gene expression, function and pathology are aligned in zone and stripe patterns [1,2,14,16,30]. The pattern of gene expression in different cerebellar lobules indicates different insult vulnerability levels. Pc death is a topographically-regulated process rather than a random sequence of events in the cerebellum [32] and it is a complex progression in neurodevelopmental disorders [2,32,33]. In the cerebellum, several studies involving mutant mice have shown that there is a general spatio-temporal pattern of Pc degeneration. These models include mouse models of Niemann-Pick type C disease [33], the lurcher mutant mouse cerebellum [34] and the ataxic sticky mouse cerebellum [35]. In most cases, specific Pc populations, which are located within the AZ and PZ, degenerate earlier than those from the CZ and NZ. It has been shown that Pc degeneration usually appears in a strip-like pattern that corresponds to a specific region within the cerebellum [32]. In murine models of Niemann-Pick disease type C, Pc loss first occurs within the AZ and PZ, and then progresses to the CZ Pcs, in which HSP25 is expressed in pattern parasagittal stripes. The Pcs that express HSP25 are more resistant to degeneration than those lacking this protein [33,34].

We recently have demonstrated that although the *nax* cerebellar cortex contains a high number of cleaved caspase-3 immuno-positive cells, none of these cells were identified as Pcs suggesting that classic apoptosis does not account for loss of Pcs in the *nax* mutant [2]. Given that the Pc degeneration is usually reflecting the fundamental architecture in cerebellum, in *nax* cerebellum either due to Pcs degeneration or developmental impairment, the number of zebrin II stripes is decreased in AZ.

Numerous genes are expressed uniformly or are entirely immune-negative in the CZ and NZ of mouse cerebellum (e.g., [1,16,36,37,38]). In contrast, a few genes such as HSP25 have been reported to be expressed in pattern parasagittal stripes in the CZ and NZ [2,30]. Surprisingly, in this study p75NTR pattern expression seems to be aligned with the HSP25 expression pattern in the CZ and NZ of the adult mouse cerebellum. It has been shown that a subset of Pcs that are HSP25 immuno-negative are more vulnerable than HSP25 immuno-positive Pcs in the CZ and NZ, which suggests that HSP25 may have a protective role in these subsets of Pcs [2,39]. HSP25 function is unclear in the cerebellum, but in non-neuronal cell lines, it is a molecular chaperone (e.g., [40]) that regulates actin filament organization and stabilization under oxidative stress challenge (e.g., [41]), regulates anti-oxidative activity [42], protects cells and increases cell survival [32]. Although the function and role of the p75NTR expression patterns in parasagittal stripes in the CZ and NZ remain to be determined, this may indicate that p75NTR in adult cerebellum accompanied by HSP25 plays a role in protection of this subset of Pcs from death. This speculation is promising because Pcs in these stripes are resistant to death in numerous neurodegenerative mutant cerebella, but there is no remarkable difference in the p75NTR expression pattern between the *nax* and wt sibling cerebella, even though there is abundant Pc death in the severed abnormal *nax* mutant cerebellum. Large Pc size, extensive connectivity and high metabolic demand lead to high vulnerability of Pcs and these neurons are specifically lost in various neurodegenerative diseases, but the molecular mechanisms underlying Pc death is poorly understood.

It has been shown that NGF likely interacts with the tyrosine kinase receptor p75NTR [43]. In addition, it is believed that cells (including neurons) are committed to die unless survival factors (such as neurotrophins) inhibit cell death through its receptors [22]. However, neurotrophin withdrawal is related to an active signal of cellular death that is induced by unbound dependence receptors [22]. These results are in agreement with recent studies indicating that small, non-peptide p75NTR ligands can selectively modify pro-survival and repair functions [25]. Given that there is no caspase-3 activation in degenerating Pcs in the *nax* cerebellum, this may imply that Pc death occurs through other death mechanism pathways [2]. Recently, it has been suggested that autophagy is involved in Pc death as a result of trophic factor deprivation through p75NTR, which is a possible mediator of Pc death [24]. Therefore, upregulation and/or downregulation or pattern and protein expression changes in p75NTR are expected under pathological conditions such as in the *nax* cerebellum because of dysregulation of pathways for numerous molecular processes and the necessity of reactions for neuronal death protection or recovery.

The pattern of p75NTR expression between the *nax* and wt sibling cerebellum was similar in immunohistochemistry and its protein expression was not significantly different during postnatal cerebellar development. Given that the HSP25 expression pattern plays a protective role in Pc death in the cerebellum of neurodegenerative mutant mice, p75NTR expression is expected to be comparable. Indeed, p75NTR protein expression in the *nax* cerebellum aligns with this speculation, and p75NTR expression is in the parasagittal stripe pattern in the CZ and NZ in *nax* and wt siblings, and it seems indistinguishable to HSP25 pattern expression. P75NTR protein expression in western blot analysis shows some upregulation, but these differences are not statistically significant between *nax* and wt. However, severe abnormality occurs in the AZ accompanied by Pc death, and our data shows that the majority of Pcs in the AZ do not express the p75NTR in adult cerebellum. More experiments are required to explore the complex functions of p75NTR expression in the cerebellum and possible protective roles that may be involved in Pc death in the *nax* cerebellum. The complexity of Pc death indicated that these large cells use different mechanisms to survive and protect their function, and therefore, different molecular pathways may trigger cell death in Pcs in pathological conditions such as the *nax* cerebellum.

## 5. Conclusions

We investigated p75NTR pattern and protein expression in the *nax* mouse with severe cerebellar anomalies and Pc degeneration and in the wt sibling cerebellum. P75NTR expression is comparable with the HSP25 expression pattern in the adult mouse cerebellum. In the *nax* cerebellum, the p75NTR pattern is similar to that of HSP25 and there is no significant difference between *nax* and wt siblings in p75NTR protein expression.
